# tANCHOR cell-based ELISA approach as a surrogate for antigen-coated plates to monitor specific IgG directed to the SARS-CoV-2 receptor-binding domain

**DOI:** 10.1093/biomethods/bpae001

**Published:** 2024-01-19

**Authors:** Hubert Bernauer, Josef Maier, Norbert Bannert, Daniel Ivanusic

**Affiliations:** ATG:biosynthetics GmbH, 79249 Merzhausen, Germany; ATG:biosynthetics GmbH, 79249 Merzhausen, Germany; Sexually Transmitted Bacterial Pathogens and HIV (FG18), Robert Koch-Institute, 13353 Berlin, Germany; Sexually Transmitted Bacterial Pathogens and HIV (FG18), Robert Koch-Institute, 13353 Berlin, Germany

**Keywords:** SARS-CoV-2, ELISA, tANCHOR, IgG antibodies, receptor binding domain (RBD), tetraspanin

## Abstract

Enzyme-linked immunosorbent assay (ELISA) systems use plates coated with peptides or expressed and purified proteins to monitor immunoglobulins derived from patient serum. However, there is currently no easy, flexible, and fast adaptive ELISA-based system for testing antibodies directed against new severe acute respiratory syndrome coronavirus 2 (SARS-CoV-2) variants. In this study, we utilized the tANCHOR protein display system that provides a cell surface decorated with the receptor-binding domain (RBD) to monitor specific antibodies derived from SARS-CoV-2 convalescent and vaccinated individuals directed against it. To test sera from vaccinees or convalescent individuals, only the RBD coding sequence needs to be cloned in the tANCHOR vector system and transfected into HeLa cells. Time-consuming protein expression, isolation, and purification followed by coating assay plates are not necessary. With this technique, the immune evasion of new SARS-CoV-2 variants from current vaccination regimes can be examined quickly and reliably.

## Introduction

Severe acute respiratory syndrome coronavirus 2 (SARS-CoV-2) caused a worldwide health crisis that began at the end of 2019 [1]. This new coronavirus was first identified in Wuhan, China, in December 2019 from a cluster of pneumonia cases. The ancestral strain was labeled Wuhan Hu-1 [2–4]. Since the first infections with the ancestral strain, several SARS-CoV-2 variants have appeared [5]. SARS-CoV-2 consists of a 30-kb single-stranded positive-sense RNA (+ssRNA) genome that encodes four structural proteins: spike (S), nucleocapsid (N), envelope (E), and membrane [6, 7]. The S protein is essential for cell entry and therefore of great interest as a vaccination target [8, 9]. The receptor-binding domain (RBD) is a part of the S1 subunit of the S protein that binds to the receptor, angiotensin-converting enzyme 2 (ACE2), for target cell infection [10]. Vaccine strategies have demonstrated that immunization with only the RBD induces neutralizing antibodies against SARS-CoV-2 [11]. Antibodies that can efficiently block the protein–protein interaction between RBD and ACE2 are considered neutralizing antibodies [12].

Mutations during replication and immune evasion occurred frequently in the genome of SARS-CoV-2 [13, 14]. Emerging SARS-CoV-2 variants escape neutralizing antibodies mainly by acquiring mutation sites within the RBD-encoding region [15]. Of note, nearly 50% of the total mutations in Omicron B.1.1.529 are in the RBD; this finding demonstrates that the RBD is important for immune escape and allows the virus to spread continuously throughout the world [16, 17]. The full S protein is not an attractive target for antibody screening against different variants because there are conserved regions, particularly the S2 region, which may result in cross-reactivity with endemic coronaviruses (CoVs) [18]. Choosing the RBD to detect antibodies against SARS-CoV-2 infection is more reliable because the RBD sequence is poorly conserved among CoVs [19].

The main SARS-CoV-2-neutralizing antibodies induced by the immune system are targeting the RBD, which is essential for cell entry [19]. Monitoring immunoglobulin G (IgG) binding to the RBD is therefore useful to test for convalescence, antibody-mediated protection, or vaccine efficiency [20]. This endeavor employs conventional enzyme-linked immunosorbent assay (ELISA) systems: A 96-well plate is coated with antigen (i.e. the RBD) [21, 22]. For each new SARS-CoV-2 variant, a new antigen must be produced to screen for antibodies that bind to the RBD. This makes it very challenging when a new ELISA system must be established or several mutations within the RBD are compared with each other. However, ELISA has become a fundamental tool to use for antibody screening. Consequently, an easy and fast adaptive ELISA-based system to test antibodies against the RBD is needed.

**Figure 1.**
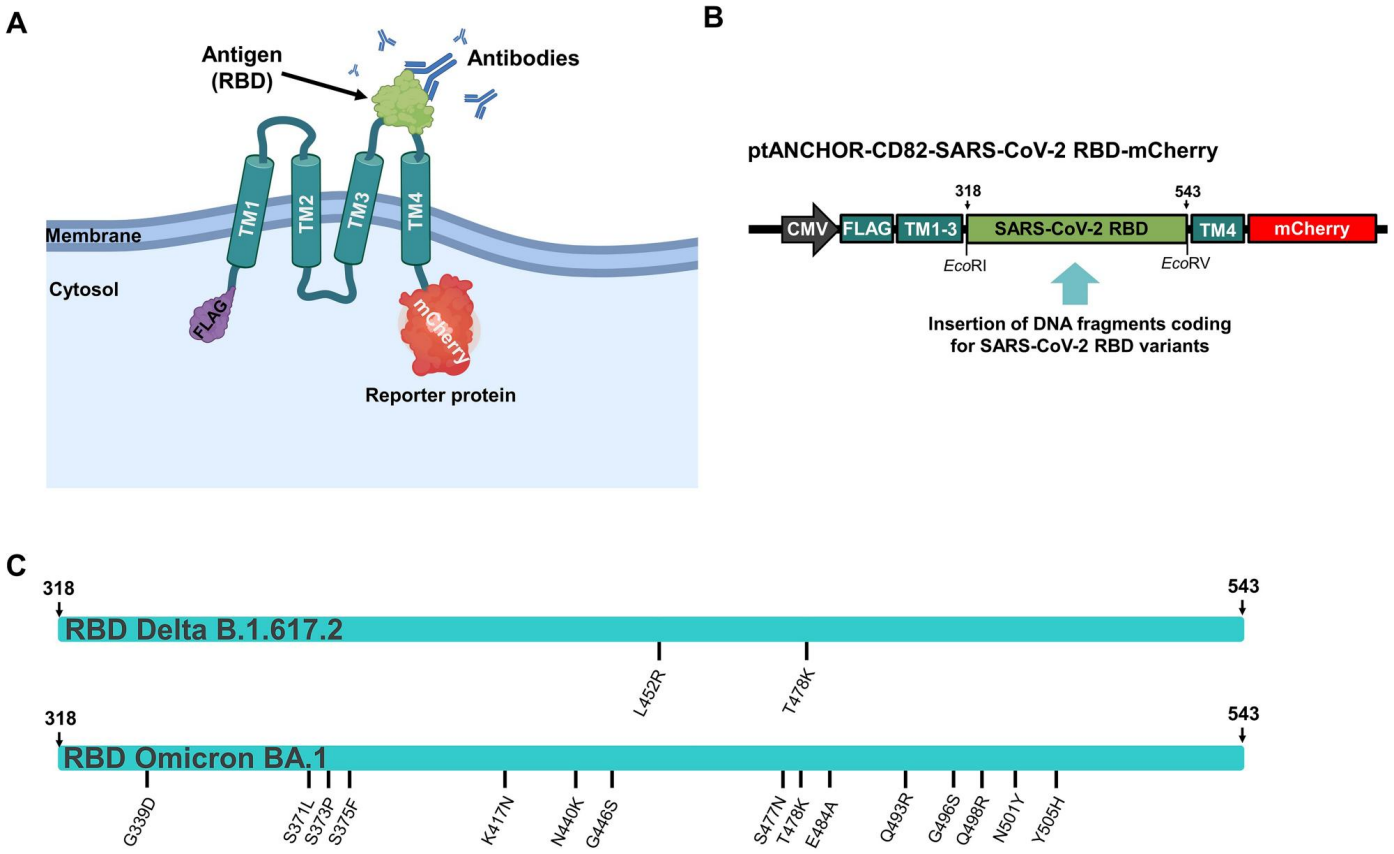
Overview of the developed cell-based IgG ELISA specific for the SARS-CoV-2 RBD. (A) Schematic representation of the topology of the expressed chimeric membrane-bound tANCHORed SARS-CoV-2 RBD and (B) the general genetic elements of the tANCHOR expression construct. TM: transmembrane domains 1–4 derived from CD82; mCherry: C-terminal red fluorescent protein mCherry used as an expression reporter protein; CMV: cytomegalovirus promoter; FLAG: N-terminal tag DYDDDK. DNA coding for the SARS-CoV-2 RBD variants was inserted in the tANCHOR vector by using *Eco*RI and *Eco*RV restriction sites to form the ptANCHOR-CD82: tANCHOR system vector backbone. (C) Illustration of the introduced mutation sites within the Delta and Omicron RBD variants. The numbers refer to the amino acid positions of the spike protein

We developed a cell-based IgG RBD-specific ELISA system to avoid the need to express, purify, and coat ELISA plates with the RBD antigen from future strains. This ELISA system is based on the tANCHOR display system, which is suitable to present proteins on the surface of human cells [23, 24]. The tANCHOR system is based on the use of tetraspanin-derived protein sequences without the large extracellular loop (LEL). The protein of interest to display on the cell surface is inserted instead of the LEL and anchored by the four transmembrane bundles (4TM) on the cell surface. Both small and large protein sequences, such as the cyan fluorescent protein (CFP), can be displayed with the tANCHOR system without affecting the protein’s fluorescent brightness [23]. We utilized the tANCHOR technology to display the RBD from various SARS-CoV-2 variants as a chimeric membrane-bound protein on the surface of HeLa cells. This approach provides extracellular accessibility for neutralizing antibodies (Fig. 1A) and allowed us to establish a rapidly adaptive IgG RBD-specific cell-based ELISA system for future SARS-CoV-2 variants.

## Materials and methods

### Molecular cloning

The generated expression constructs to display the RBD of SARS-CoV-2 on the cell surface are based on the tANCHOR display system [23]. Inserts encoding the RBD of Wuhan (Wuhan-Hu-1), Delta (B.1.617.2), and Omicron (BA.1) were introduced into the vector ptANCHOR-CD82-V5-6xHis-mCherry (ATG:biosynthetics GmbH, Merzhausen, Germany) by using the restriction sites *Eco*RI and *Eco*RV. The DNA sequences of the RBD inserts (Supplementary Table 1) were obtained from the GISAID database [25] and produced by gene synthesis (ATG:biosynthetics GmbH). The vector ptANCHOR-CD82-Wuhan-RBD-YFP was generated by exchanging the mCherry coding sequence with the YFP DNA fragment amplified by using the primers YFP-*Cla*I for and YFP-*Pme*I rev (Supplementary Table 2) and the template vector pCMV-CD63-YFP [26]. The YFP fragment was digested with *Cla*I and *Pme*I and ligated into the restricted vector ptANCHOR-CD82-Wuhan-RBD-mCherry. Oligonucleotides were purchased from Integrated DNA Technologies (Leuven, Belgium), and the vector pmCherry-N1 was procured from Clontech (Heidelberg, Germany). Vector sequences were confirmed by restriction analysis and Sanger sequencing (Supplementary Information protocol for Sanger sequencing). Plasmid DNA for transfection was isolated from *Escherichia coli* DH5α (New England Biolabs, Frankfurt, Germany) by using the Plasmid Maxi Kit (Qiagen, Hilden, Germany) following the manufacturer’s instructions (Supplementary Information protocol for isolation of plasmid DNA). Plasmid DNA concentration was measured by using a NanoVue spectrophotometer (GE HealthCare, Solingen, Germany).

### Cell-based SARS-CoV-2 RBD-specific IgG ELISA

HeLa cells were maintained in Dulbecco’s Modified Eagle’s Medium (DMEM) that was supplemented with 2 mM l-glutamine, 10% fetal bovine serum (FBS, Gibco, Langenselbold, Thermo Fisher Scientific, Germany), 100 U mL^−1^ penicillin, and 100 µg mL^−1^ streptomycin, and incubated at 37°C in humidified air containing 5% CO_2_. First, 1.5 × 10^4^ HeLa cells per well of a 96-well cell culture plate (TPP, Trasadingen, Switzerland) were seeded. After the cells reached ∼80% confluency (typically overnight), they were transfected with 0.3 µg of the indicated plasmid DNA and 1 µL of Metafectene (Biontex, Munich, Germany) according to the manufacturer’s instructions (Supplementary Information protocol for cell transfection mix). The transfection mix was incubated for 24 h, and then the medium was replaced with 100 µL of fresh medium containing FBS, and the cells were incubated for additional 24 h. Next, the cells were washed once with 450 µL of 1× phosphate-buffered saline (PBS) and then fixed for 15 min with 2% paraformaldehyde (PFA, Carl Roth, Karlsruhe, Germany) in 1× PBS. After fixation, the cells were washed twice with 450 µL of 1× PBS and then incubated for 1 h with freshly prepared blocking buffer containing 2% chicken egg albumin (CEA) (Sigma-Aldrich, Steinheim, Germany), 3% bovine serum albumin (BSA) fraction V (Carl Roth), and 10% normal goat serum (NGS) or normal rabbit serum (Biowest, Nuaillé, France) if indicated. The cells were then incubated with diluted sera in blocking buffer for 1 h, followed by five washes with 450 µL of wash buffer (1× PBS containing 0.05% Tween-20). To detect bound human IgG on the RBD-displaying target cells, they were incubated with 100 µL of anti-human IgG conjugated to horseradish peroxidase (HRP) diluted 1:8000 (1.3 mg/mL, P0214, Dako, Hamburg, Germany) for 30 min in blocking buffer. After incubation, the cells were washed five times with washing buffer. Antibodies bound by anti-human IgG conjugated to HRP were detected by incubation with 80 µL of the HRP substrate 3,3′,5,5′-tetramethylbenzidine (TMB, Bio-Rad Laboratories GmbH, Feldkirchen, Germany) for 15 ± 2 min. The enzyme reaction was stopped by adding 100 µL of 2 M H_2_SO_4_ to each well of the 96-well plate. Absorbance was measured at a 450 nm with a reference wavelength of 620 nm by using a spectrophotometer (Tecan, Crailsheim, Germany). All washing cycles were performed using the BioTec 405 ELISA washer (Agilent Technologies, Waldbronn, Germany) with the following settings: a volume of 450 µL, the lowest flow rate of 3, and a 30-s delay time. The cut-off was calculated by using the mean plus three standard deviations (SDs) derived from the negative controls.

### S1- and nucleocapsid protein-specific ELISA

A ready-to-use coated ELISA plate from the Anti-SARS-CoV-2 (IgG) kit (EUROIMMUN, Lübeck, Germany) was used to test IgG binding to the Wuhan S1 protein. This testing was performed in parallel to cell-based ELISA with the same method (the TMB reaction was stopped after 5 min). To confirm SARS-CoV-2 convalescence for individual serum samples, 96-well MaxiSorp plates (Thermo Fisher Scientific) were coated with 100 µL of 0.5 µg mL^−1^ SARS-CoV-2 NCP containing the amino acids 2-419 (Miltenyi Biotec, Bergisch Gladbach, Germany) diluted in coating buffer (15 mM Na_2_CO_3_, 35 mM NaHCO_3_, pH 9.6) and incubated overnight sealed with ROTILABO sealing film (Carl Roth) at 4°C. The plate was then washed three times with 1× PBS, incubated with 100 µL of blocking buffer containing 2% CEA and 3% BSA fraction V for 30 min, washed three times with 1× PBS, and incubated with 100 µL of diluted sera in blocking buffer containing 10% NGS. After incubation for 1 h, the plate was washed five times with 450 µL of wash buffer (1× PBS containing 0.05% Tween-20). Then, 100 µL of anti-human IgG conjugated to HRP (Dako) diluted 1:5000 in blocking buffer containing 10% NGS was added to each well; the plate was incubated for 45 min. After incubation with the secondary antibody, the plate was washed five times with 450 µL of wash buffer. HRP activity was detected by adding 80 μL of TMB (Bio-Rad Laboratories) and incubating the plate at room temperature for 5 min. The reaction was stopped by adding 100 μL of 2 M H_2_SO_4_. Absorbance was measured at 450 nm with background correction at 620 nm by using a Tecan Infinite 200 microplate reader. The cut-off was calculated for all performed ELISA by using the mean plus three standard deviations (SDs) obtained from the absorbance values of the negative controls.

### SARS-CoV-2 RBD-specific ELISA

To screen for SARS-CoV-2 RBD-specific IgG, Nunc Maxisorp plates (Thermo Fisher) were coated with 50 µL of 3 µg/mL Wuhan, Delta, and Omicron RBD protein (Proteogenix, Schiltigheim, France) diluted in coating buffer (15 mM Na_2_CO_3_, 35 mM NaHCO_3_, pH 9.6). The assay plates were sealed with ELISA sealing film (Carl Roth) and incubated overnight at 4°C. Then, the plates were washed three times with 400 µL of wash buffer and incubated for 1 h with blocking buffer. After blocking, human sera containing IgG antibodies were diluted in blocking buffer and incubated for 1 h, followed by five washes with 450 µL of wash buffer (1× PBS containing 0.05% Tween-20). All other steps were performed as described for the cell-based ELISA approach.

### Analysis of bound human anti-RBD IgG by confocal laser microscopy

HeLa cells (1 × 10^4^ per well) were seeded onto a high glass-bottomed 8-well μ-slide (Ibidi, Munich, Germany). Twenty-four hours later, the cells were transiently transfected with 0.3 μg plasmid DNA and 1 μL of Metafectene. The transfection mix was incubated for 24 h, and then the medium was replaced with 200 µL of fresh medium containing FBS, and the cells were incubated for additional 24 h. After 48 h, the cells were washed once with 1× PBS and fixed with 200 μL of 2% PFA in 1× PBS for 20 min. The cells were washed once with 1× PBS. Pooled sera from convalescent individuals diluted 1:200 in 3% BSA and 2% CEA was added to each well, and the plate was incubated at room temperature for 1 h. The cells were washed three times. Then, to detect bound IgG, the cells were incubated with goat anti-human IgG conjugated to Alexa 488 (A-11013, 2 mg/mL, Thermo Fisher) diluted 1:3000 in 3% BSA and 2% CEA for 1 h. After washing three times, the cells were left in 200 μL of 1× PBS with 1 μL of Hoechst 33342 staining solution (ImmunoChemistry Technologies, Davis, CA, USA). Images were acquired with an inverted confocal laser scanning microscope (LSM 780; Carl Zeiss Microscopy GmbH, Oberkochen, Germany) and a plan-apochromat oil-immersion objective lens (63×, numerical aperture 1.4; Carl Zeiss Microscopy GmbH). Fluorescence signals were detected with the Zeiss ZEN smart setup settings for Hoechst 33342 (excitation at 405 nm and emission at 464 nm [429–499 nm], 0.8% laser power), Alexa 488 (excitation at 488 nm and emission at 546 nm [493–598 nm], 1.0% laser power), and mCherry (excitation at 594 nm, emission at 648 nm [599–696 nm], 8.0% laser power). The slides were scanned unidirectionally with a fixed pixel size of 0.11 μm (1024 × 1024 pixels per image).

### Analysis of tANCHORed Delta and Omicron RBD co-localization with Wuhan RBD

HeLa cells (1 × 10^4^) were seeded onto each well of a high glass-bottomed 8-well μ-slide (Ibidi). Twenty-four hours later, the cells were transfected in the same way as described in the section for analysis of bound human anti-RBD IgG by confocal laser microscopy. Then, the cells were fixed with 200 μL of 2% PFA in 1× PBS for 20 min. After fixation, the cells were left in 200 μL of 1× PBS. Images for co-localization analysis were acquired with an inverted LSM 780 confocal laser scanning microscope and a plan-apochromat oil-immersion objective lens (63×, numerical aperture 1.4; Carl Zeiss Microscopy GmbH). The fluorescence signals emitted by mCherry and yellow fluorescent protein (YFP) were detected with the Zeiss ZEN smart setup settings for YFP (excitation at 514 nm, emission at 551 nm [516–586 nm], 1.5% laser power) and mCherry (excitation at 594 nm, emission at 648 nm [599–696 nm], 6.5% laser power), respectively. Control between the displayed Wuhan RBD and the nucleus was stained with Hoechst 33342 dye. Fluorescence signals emitted by Hoechst 33342 and YFP were detected with the settings for Hoechst 33342 (excitation at 405 nm, emission at 466 nm [426–506 nm], 0.6% laser power) and YFP (excitation at 514 nm, emission at 551 nm [516–586 nm], 1.2% laser power). The slides were scanned unidirectionally with a fixed pixel size of 0.14 μm (512 × 512 pixels per image). Pearson correlation coefficients (PCCs) were calculated by employing the Fiji ImageJ Coloc2 plugin.

### Quantification of expressed proteins fused to mCherry

The expressed tANCHORed proteins contain a C-terminal mCherry reporter protein that was used for quantification via the mCherry quantification kit (Abcam, Cambridge, UK). HeLa cells were transfected in the same way as described above for a 96-well plate. After 48 h, HeLa cells were lysed in 35 µL per well of mCherry assay buffer and incubated on ice for 15 min. Cell lysates from a pool of three wells were collected in a 1.5-mL reaction tube. Cell debris was removed by centrifugation, and 100 µL of the supernatant was transferred to a 96-well optical plate (Greiner Bio-One, Frickenhausen, Germany). The mCherry standard curve used to quantify mCherry fusion proteins was constructed by diluting the mCherry standard protein. All fluorescence values were measured using the GloMax-Multi+ detection system and the filter kit green for red dyes (Promega, Fitchburg, WI, USA). From all fluorescence signal readings, the relative light unit (RLU) value measured for wells containing only mCherry assay buffer was subtracted.

### Human serum material

Serum panel samples from infected individuals with a polymerase chain reaction (PCR)-confirmed SARS-CoV-2 infection were collected between March 2021 and February 2022 during hospitalization at the University Medical Center, Freiburg (*n* = 13). Sera were also obtained from people who had received one (*n* = 5), two (*n* = 10), or three (*n* = 10) doses of the BNT162b mRNA vaccine. Serum samples from anonymous healthy controls (negative), who had tested negative for SARS-CoV-2 IgG, were used as negative controls (*n* = 10).

### Statistical analysis

The Shapiro–Wilk test was used to determine whether the data followed a Gaussian distribution. The data are presented as the mean ± SD. Student’s unpaired *t* test was used to compare between two groups by using Graph Pad Prism Version 9.2.0 (GraphPad Software, San Diego, CA, USA). A *P* < .05 was considered to be statistically significant. Asterisks are used to indicate statistical significance in the figures: *P < .05; **P < .01; ***P < .001; ****P < .0001; not significant (ns) *P* > .05.

### Digital illustrations

Images and illustrations were created with BioRender.com, Microsoft Office 2016, and Graph Pad Prism version 9.2.0.

## Results

### Generation of expression constructs for anchoring the RBD of SARS-CoV-2 to the cell surface

We used the previously developed tANCHOR display system [23] to generate a reactive cell surface for antibodies directed against the RBD. This display system utilizes a CD82 tetraspanin anchor where the inserted sequence is displayed between transmembrane helices three and four, providing extracellular accessibility to anti-RBD antibodies (Fig. 1A). By cloning gene synthesis fragments (flanked with the *Eco*RI and *Eco*RV sequences), we were able to insert DNA sequences encoding the Wuhan-Hu-1 RBD (amino acids 318–543) as well as sequences encoding the Delta (B.1.617.2) and Omicron (BA.1) RBD variants (Fig. 1B and C).

### Protein expression analysis of the generated expression constructs

**Figure 2.**
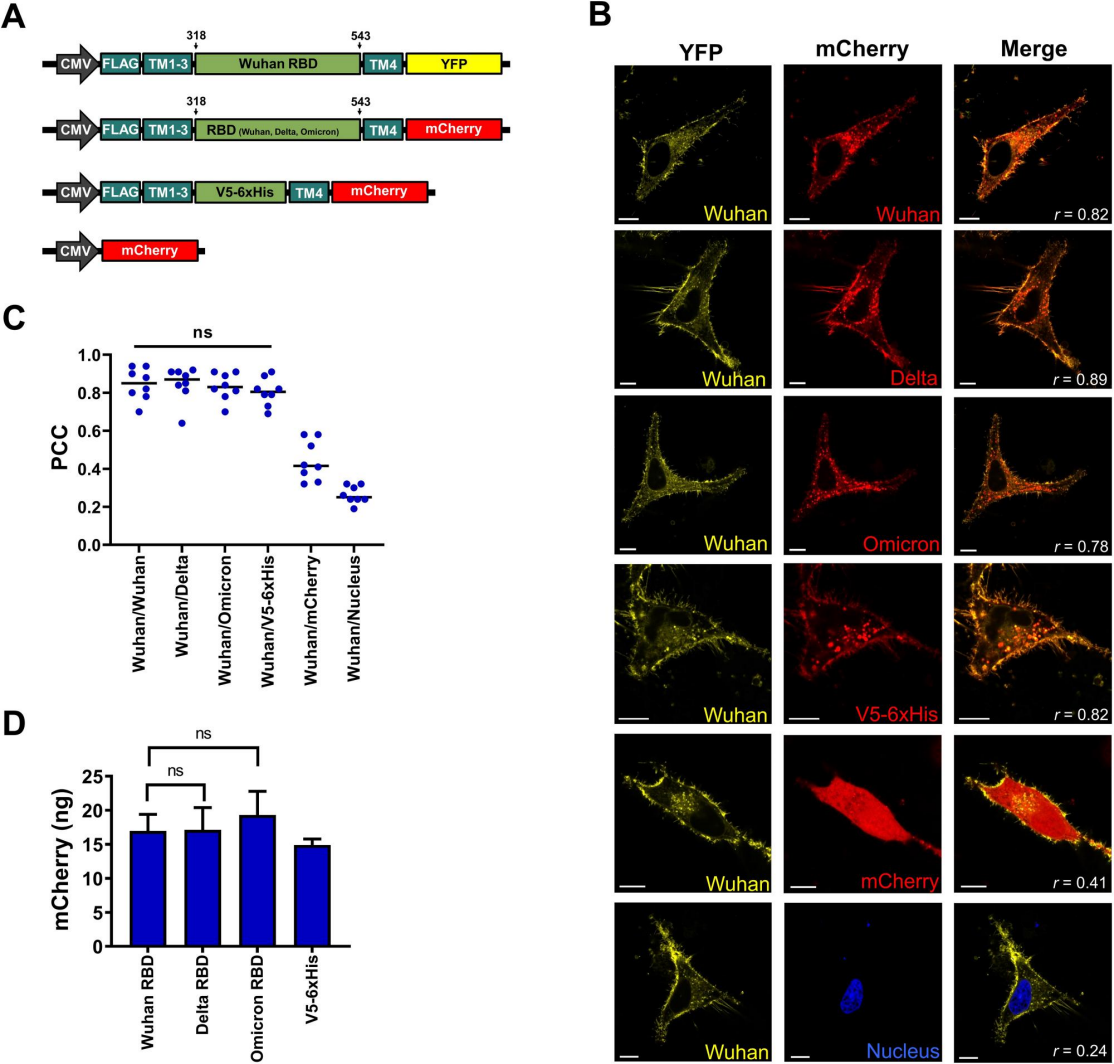
Protein expression analysis of the generated constructs. (A) Illustration of the expression constructs used for protein distribution and expression analysis of the receptor-binding domain (RBD) variants. V5-6xHis: V5 peptide epitope tag of the simian virus 5 (GKPIPNPLLGLDST) and 6x histidine tag; YFP: yellow fluorescent protein; FLAG: N-terminal tag DYDDDK; TM: transmembrane domain 1–4 derived from CD82. The numbers refer to the amino acid positions of the spike protein. (B) Representative confocal laser micrographs showing co-localization of the expressed tANCHORed Wuhan RBD with the Delta and Omicron RBD variants. Monomeric mCherry for cytosolic protein distribution was expressed using the vector pmCherry-N1, and for surface distribution the vector ptANCHOR-CD82-V5-6xHis-mCherry. Nuclei were stained with Hoechst 33342. The scale bars are 10 µm. (C) The Calculation of Pearson correlation coefficients showed no significant differences regarding protein distribution within the cell between the RBD variants and the Wuhan RBD (*n* = 10). Significance was determined with a one-way analysis of variance (non-significant [ns], *P* > .05). (D) Quantification of mCherry-fused tANCHORed RBD (*n* = 4). The *P*-value was calculated by using an unpaired two-tailed Student’s *t* test (ns, *P* > .05). The control was used to express a tANCHORed V5-6xHis sequence.

We performed co-localization experiments to analyze putative differences in protein distribution within the cell. We compared the protein distribution of tANCHORed RBD variants fused with mCherry with the ancestor RBD (Wuhan-Hu-1), which was fused with yellow fluorescent protein (YFP) (Fig. 2A). Both fluorescence signals emitted by mCherry and YFP can be separated, and we monitored the co-localization by using a two-channel image. As controls, we used expressed tANCHORed V5-6xHis tag (confirmed cell surface presentation [23, 27]) and mCherry (for cytoplasmatic distribution). In addition, we stained the nuclei with Hoechst 33342 as a negative control because we did not expect protein localization within the nucleus. We observed predominant cell surface localization for the tANCHORed RBD as well as the displayed V5-6xHis tag (Fig. 2B). The calculated PCCs indicated a significant and strong correlation between the displayed Wuhan RBD fused to YFP and the Delta RBD as well as the Omicron RBD fused to mCherry. The obtained PCCs are comparable to the V5-6xHis tag control (Fig. 2C). This finding confirmed that there is no significant difference in protein distribution when the RBD sequence contains mutations present in the Delta or Omicron variant (Fig. 2C). Next, we quantified the protein expression by utilizing the mCherry reporter protein (Fig. 2D). We did not observe significant protein expression differences in the expression constructs containing the Wuhan, Delta, and Omicron RBD variants, and all were localized predominantly at the cell surface.

### Optimizing blocking conditions to reduce nonspecific antibody binding to HeLa cells

**Figure 3.**
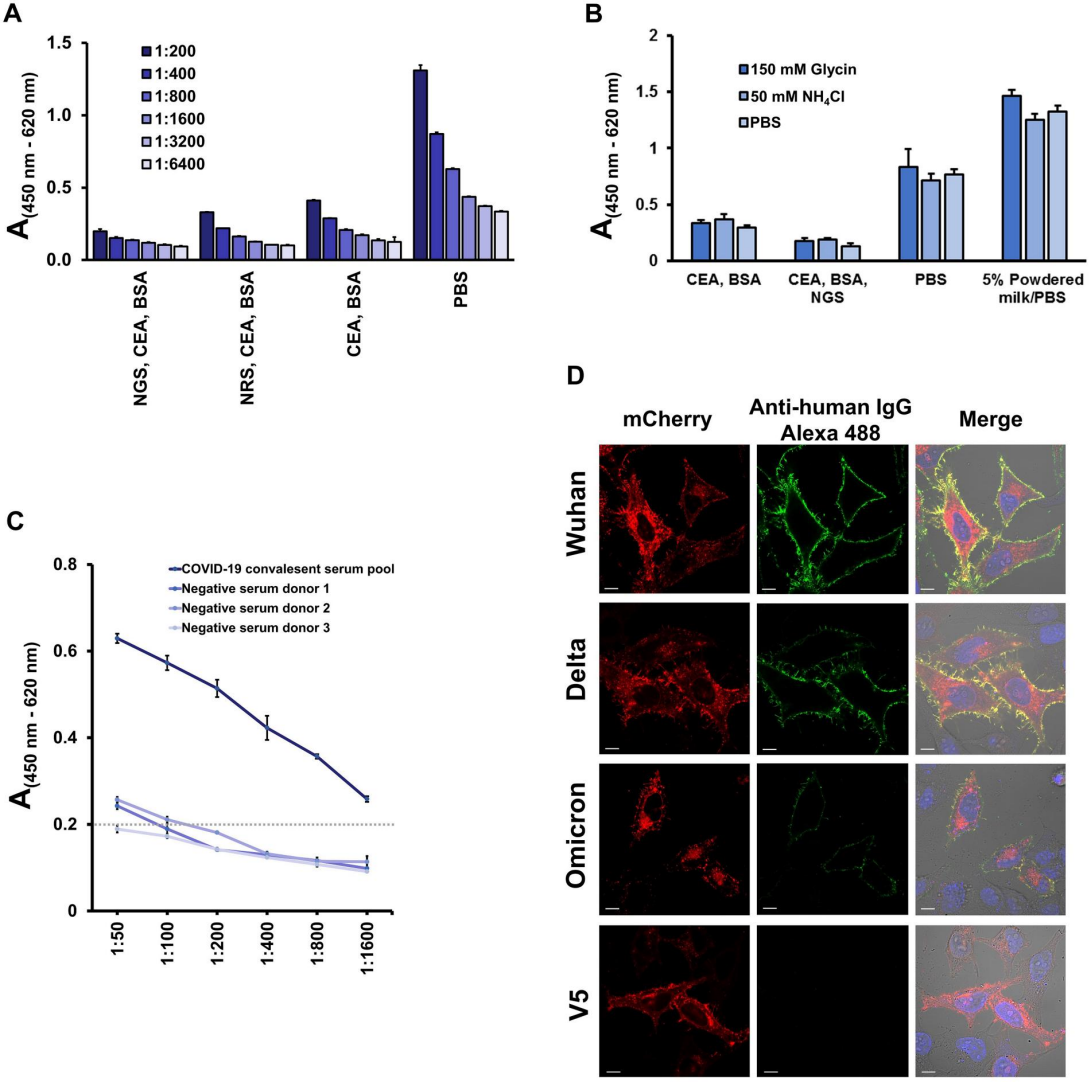
Optimization of the developed tANCHOR cell-based ELISA. (A) Testing of different blocking conditions using HeLa cells incubated for 1 h with COVID-19-negative serum diluted 1:200. Blocking conditions were investigated by using protein-based blocking agents and (B) reagents to block unreacted aldehydes after fixation with paraformaldehyde. BSA: bovine serum albumin (3%); CEA: chicken egg albumin (2%); NGS: normal goat serum (10%); NRS: normal rabbit serum (10%); PBS: 1× phosphate-buffered saline. (C) Dilution-dependent IgG binding of serum from the COVID-19 convalescent group and COVID-19-negative donors on HeLa cells transfected with the plasmid ptANCHOR-CD82-RBD-Wuhan-mCherry. The broken line indicates an absorbance value of 0.2. A: absorbance measured at 450 nm (reference wavelength 620 nm). (D) Representative confocal laser micrographs of HeLa cells transfected with the indicated expression constructs and tested for IgG binding from the pooled serum from the COVID-19 convalescent group. Bound IgG antibodies were detected with goat anti-human IgG conjugated to Alexa 488. The control contains HeLa cells transfected with the vector ptANCHOR-CD82-V5-6xHis-mcherry and was treated in parallel and in the same way as other samples. The scale bars are 10 µm.

The major challenge when performing a cell-based ELISA is to reduce the nonspecific binding of antibodies to the cell surface. We tried different blocking recipes and found that using a freshly prepared solution containing 3% BSA, 2% CEA, and 10% NGS diluted in 1× PBS resulted in the highest reduction of nonspecific binding of antibodies derived from coronavirus disease of 2019 (COVID-19) negative serum to the surface of HeLa cells (Fig. 3A). In contrast, without a blocking buffer, nonspecific binding resulted in a high absorbance value (> 1.3). In addition, we tested whether it would be possible to reduce the background further by blocking unreacted aldehydes after fixing the cells with PFA [28–30]. We incubated cells after the fixation step with 150 mM glycine or 50 mM ammonium chloride dissolved in 1× PBS and blocked with the optimized blocking buffer. Preincubation with glycine or ammonium chloride did not decrease the background absorbance more than preincubation with only 1× PBS (Fig. 3B). This result indicates that blocking for 1 h with 3% BSA, 2% CEA, and 10% NGS in 1× PBS is the best blocking method for the cell-based RBD-specific ELISA. Interestingly, blocking with 5% low-fat powdered milk (blotting grade) increased the nonspecific background binding and is therefore inappropriate (Fig. 3B). Next, we diluted serum derived from a COVID-19 convalescent pooled group and three COVID-19-negative donors. We found that a serum dilution of 1:200 in blocking buffer is able to keep the background absorbance at < 0.2 (Fig. 3C). For this reason, we chose a 1:200 dilution of serum as the optimum dilution in other performed ELISA. Considering the optimized conditions, we tested IgG binding to HeLa cells transfected with the indicated tANCHOR constructs. We observed only strong binding of IgG, detected with the secondary goat anti-human IgG conjugated to Alexa 488, for HeLa cells that had been transfected with tANCHOR vectors containing the Wuhan or Delta RBD coding sequence. In contrast, on the cell surface with the displayed Omicron RBD, we only detected a fraction of the IgG (Fig. 3D). We did not detect IgG binding on HeLa cells that displayed the V5-6xHis tag. This visual experiment using confocal laser scanning microscopy showed that it is possible to detect differences in antibody binding on the displayed RBD protein.

### Testing IgG binding of sera from a COVID-19 convalescent group, vaccinated individuals, and COVID-19-negative donors to transfected HeLa cells

**Figure 4.**
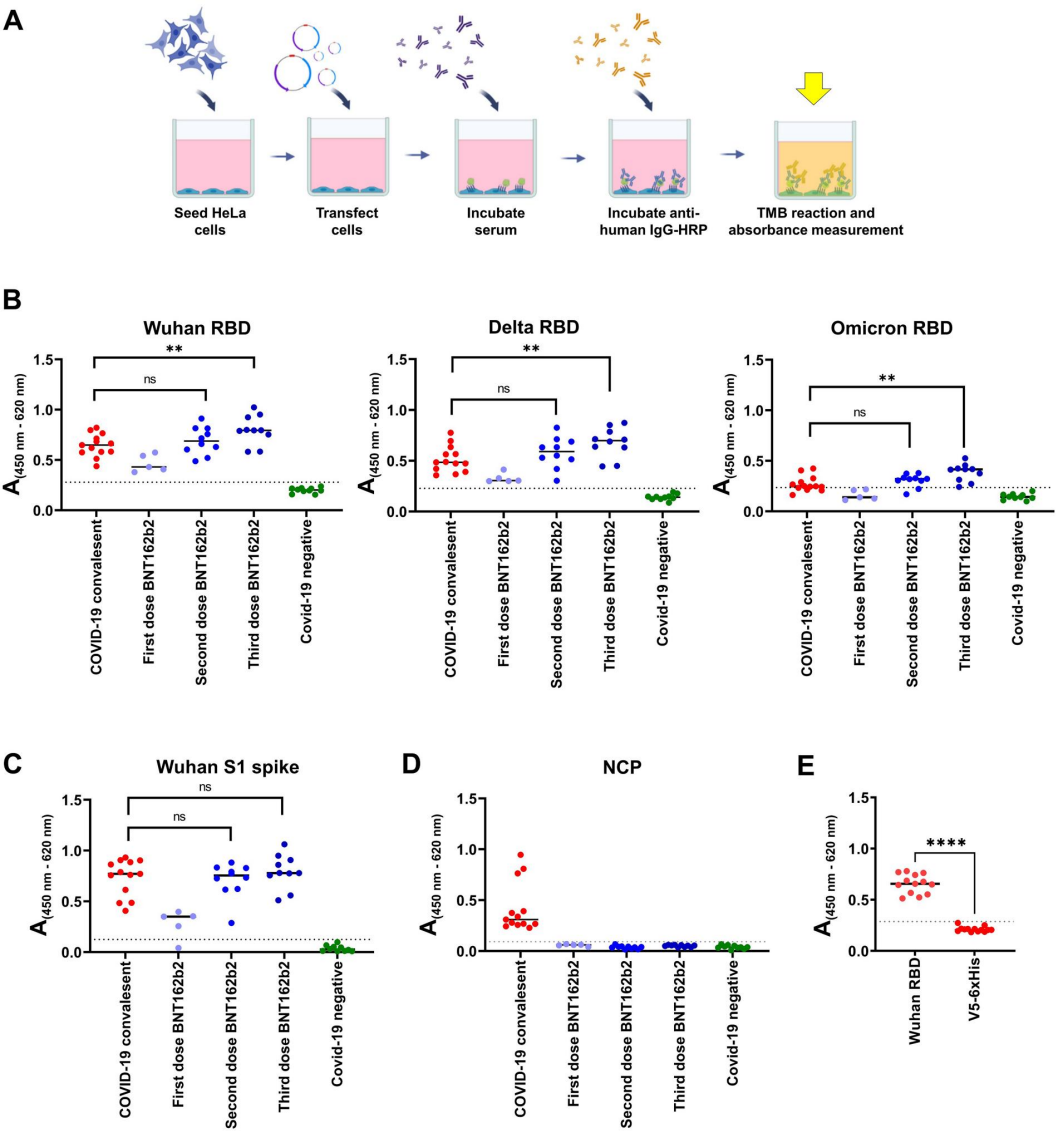
Cell-based ELISA for measuring anti-SARS-CoV-2-RBD-specific immunoglobulin (IgG). (A) A cartoon shows the schematic workflow for measuring IgG against the SARS-CoV-2 RBD. (B) HeLa cells were transfected with the indicated vectors ptANCHOR-CD82-Wuhan-RBD-mCherry, ptANCHOR-CD82-Delta-RBD-mCherry or ptANCHOR-CD82-Omicron-RBD-mCherry. Absorbance (A) obtained from the cell-based ELISA specific for IgG antibodies directed against the indicated RBD. (C) Testing for IgG binding on the EUROIMMUN ELISA plate coated with the SARS-CoV-2 S1 protein. (D) Validation of the serum panel for reactivity with the SARS-CoV-2 nucleocapsid protein (NCP) by using an ELISA plate coated with amino acids 2-419 of the NCP. (E) Detection of IgG antibodies against SARS-CoV-2 derived from convalescent individuals binding on RBD displayed Wuhan RBD and the control epitopes V5-6xHis. Each plotted A value was measured in duplicate or triplicate (E) at 450 nm (reference wavelength: 620 nm). The *P*-value was calculated with an unpaired two-tailed Student’s *t* test (**P < .01; ****P < .0001; not significant [ns], *P* > .05). The broken line in the graph represents the cut-off value.

Our test of antibody binding to transfected HeLa cells is based on previous observations: We showed that the use of HeLa cells results in a stable cell layer after washing steps [23]. The complete workflow for a cell-based ELISA can be performed within 3 days. First, cells are seeded in a 96-well plate and transfected with the generated tANCHOR vectors for displaying the Wuhan, Delta, and Omicron RBD on the cell surface. Forty-eight hours later, antibodies can be added to see if they bind to the desired antigen on the cell surface. Antibodies that bind to the displayed antigen can be detected by incubation with an HRP-conjugated secondary antibody (e.g. anti-human IgG conjugated to HRP; Fig. 4A). By employing sera from a COVID-19 convalescent group (*n* = 13); vaccinees who had received one (*n* = 5), two (*n* = 10), or three (*n* = 10) doses of the BNT162b mRNA vaccine; and COVID-19-negative donors (*n* = 10), we demonstrated that there are different IgG binding efficiencies to the Wuhan, Delta, and Omicron RBD variants (Fig. 4B). In all ELISA experiments, we observed significant differences between the COVID-19 convalescent group and the vaccinees who had received three doses of the BNT162b mRNA vaccine. Interestingly, when we tested the same serum samples on the EUROIMMUN ELISA plate coated with the S protein, we did not observe differences between the COVID-19 convalescent pool and vaccinees who had received two or three doses of the BNT162b RNA vaccine (Fig. 4C). This is explained by the fact that neutralizing and non-neutralizing antibodies bind to the S1 protein. Testing antibodies directed against the nucleocapsid protein (NCP) is useful to differentiate between infected and vaccinated individuals because current vaccines are not expected to elicit an antibody response against the NCP [31]. We further ruled out that the vaccinees had been infected prior to or during vaccination with the SARS-CoV-2 virus because only the COVID-19 convalescent group was reactive for the NCP based on an NCP-specific ELISA (Fig. 4D). To ascertain whether the developed cell-based ELISA remained specific for the SARS-CoV-2 RBD, we applied the serum panel of convalescent individuals on HeLa cells transfected with the vector ptANCHOR-CD82-Wuhan-RBD-mCherry or ptANCHOR-CD82-V5-6xHis-mCherry. We observed, that there is no cross-reactivity between the displayed RBD protein and the V5-6xHis tag (Figure 4E).

### Comparison with RBD-antigen-coated plates

**Figure 5.**
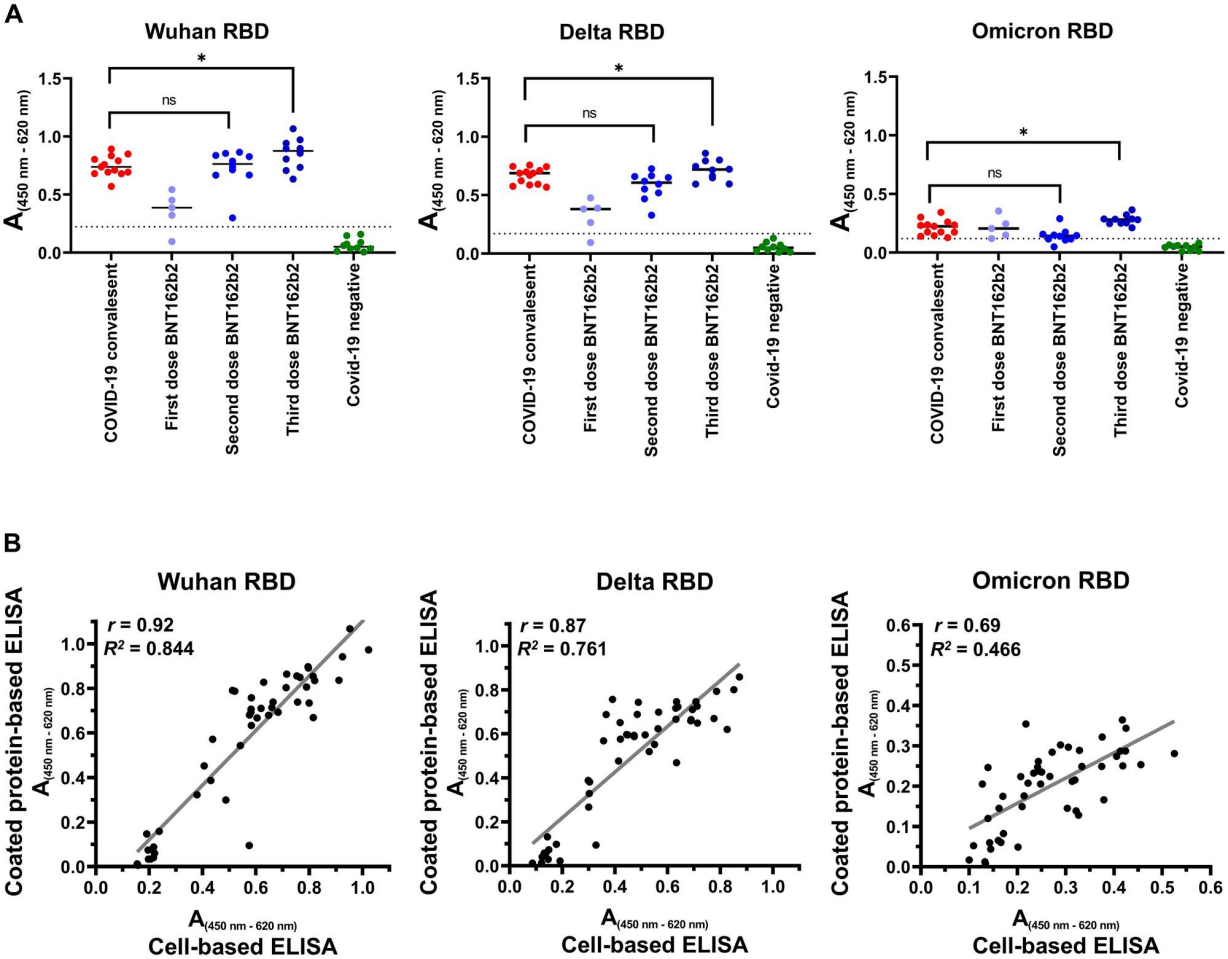
Comparison with ELISA plates coated with purified RBD protein. (A) Absorbance (A) results from testing the serum panel for IgG binding to the indicated purified recombinant RBD protein. Each plotted A value was measured in duplicate at 450 nm (reference wavelength: 620 nm). (B) Correlation of the A results obtained from ELISA plates with expressed tANCHORed RBD on a HeLa cell layer and from ELISA plates coated with purified recombinant RBD. The *P*-value was calculated with an unpaired two-tailed Student’s *t* test (*P < .05; not significant [ns], *P *>* *.05). The broken line in the graph represents the cut-off value.

We compared the developed tANCHOR-based ELISA with conventional protein-coated assay plates in terms of IgG binding. We carried out the experiment in parallel to the cell-based ELISA and used the same conditions (serum dilution of 1:200, incubation time, antibody concentration, and handling). We obtained similar results when we tested the serum samples on the protein-coated assay plates (Fig. 5A). Next, to assess the performance of the RBD-displaying cell layer against RBD-coated assay plates, we performed a Pearson correlation analysis. A very strong correlation was observed for the Wuhan (*r *=* *.92) and Delta (*r *=* *.87) RBD antigens with the corresponding tANCHORed RBD (Fig. 5B). However, for the Omicron RBD antigen, the correlation with the tANCHORed Omicron RBD was lower (*r *=* *.69), although it is still considered a strong correlation. This lower correlation could be the result of lower absorbance values because the serum samples did not contain Omicron-specific antibodies. Taken together, a cell layer displaying the RBD antigen is a reliable surrogate technique for recombinant RBD protein-coated assay plates.

## Discussion

Coating assay plates with recombinant protein is the first step in any conventional ELISA technique in which adsorption occurs via hydrophobic interactions of the diluted antigen to the surface of the well [32, 33]. This approach is mostly used to detect pathogen-specific antibodies in a clinical sample and provides high accuracy [34–41]. However, such ELISA systems require an isolated and purified antigen or synthesized peptide. When monitoring antibodies directed against the RBD, which is 225 amino acids, it is very common to express such antigens in bacteria or mammalian cells [42, 43]. When coating the wells of an assay plate, the conditions regarding the buffer for antigen stability, and, in particular, surface adsorbance must be optimized. The involved steps from expression until coating the assay plates can be very time-consuming, and when a new ELISA has to be developed, an alternative, fast, adaptable technique is needed. We addressed this gap by using HeLa cell layers displaying the desired antigen on the surface instead of an antigen-coated assay plate. In this study, we focused on detecting antibodies derived from clinical samples directed against the RBD of SARS-CoV-2. Although we previously demonstrated in principle that it is possible to detect purified antibodies directed against epitopes 2F5/4E10 and the immunosuppressive domain (ISU) of HIV-1 by using the tANCHOR system, it was still unclear whether clinical samples could also be screened [23, 24]. Therefore, we optimized this cell-based ELISA approach by employing the tANCHOR system to monitor IgG from clinical samples by using serum samples containing antibodies against the S protein of SARS-CoV-2.

The detection of RBD-specific IgG antibodies is very important because they are considered neutralizing antibodies for SARS-CoV-2 [44]. It is clear that for every new upcoming variant, a new antigen must be generated to measure IgG binding. The hallmark of RNA viruses is a high mutation rate, which allows them to escape neutralizing antibodies and makes the host susceptible again, a phenomenon that has been observed for the SARS-CoV-2 Omicron variant [45–47]. The tANCHORed RBD antigen display system is suitable to establish, in a fast and easy way, a new assay for any future SARS-CoV-2 variant. As we demonstrated, mutation sites for the Delta and Omicron variants did not cause protein mislocalization or influence protein expression (Figs. 2C and D). Additionally, the RBD of the Wuhan, Delta, and Omicron variants is displayed on the cell surface in a native confirmation, as our earlier study demonstrated by performing an angiotensin-converting enzyme 2 (ACE2) binding assay [48]. For that reason, the binding affinity of the RBD to ACE2 reflects a native conformation of the recombinant RBD [11]. It is advantageous to use the three-dimensional native structure of antigens in order to replicate the natural binding characteristics of antibodies [49, 50].

The most challenging and significant part of optimization is reducing the nonspecific binding of antibodies to cells. We adapted the blocking optimization that was done for proximity ligation assays—in which very low amounts of nonspecific IgG binding caused high background—and observed that a mixture of BSA and CEA alongside serum markedly reduced absorbance background [51–53]. We found that for cell-based ELISA a blocking buffer containing 3% BSA, 2% CEA, and 10% NGS was able to keep the absorbance value of background binding in our performed cell-based ELISA below or at 0.2 (Fig. 3A). In contrast, without the use of an optimized blocking buffer, the nonspecific binding increased the absorbance value to 1.3 (Fig. 3A).

It is worth highlighting that the direct comparison between the technique using RBD-coated assay plates and RBD displayed on HeLa cells showed a very strong correlation for the Wuhan (*r *=* *.92) and Delta (*r *=* *.87) RBD variants (Fig. 5B). Of note, the correlation between the Omicron RBD and tANCHORed Omicron RBD was lower compared with the Wuhan and Delta RBD variants, but it is still considered a strong correlation (*r *=* *.69). This observation can be explained by the lower absorbance values that are obtained for the weak antibody binding to the Omicron RBD. Absorbance values are more affected by inaccuracy when there is low antibody binding. As far as we are aware, this is the first report utilizing a displayed RBD of SARS-CoV-2 on the cell surface for antibody binding assays. Other cell-based assays use soluble RBD protein to test ACE2 binding on Vero cells in the presence of anti-SARS-CoV-2 antibodies [54]. Such an assay is useful for detecting neutralizing antibodies but not for monitoring SARS-CoV-2 IgG antibodies directed against the RBD. Furthermore, the lack of a method for displaying the RBD on the surface of cells has the implications that for every emerging variant, a new RBD protein has to be expressed, isolated, and purified. This could require some time if comparing several RBD variants. Displaying the RBD on the cell surface of adherent cells like HeLa enables the simple setup of a serological assay to track antibodies targeting SARS-CoV-2. There is also the possibility to compare different cell-based ELISA measurements by utilizing reference material in order to determine binding antibody units (BAU) per milliliter for each sample [55, 56]. The use of defined standard material would allow researchers to avoid comparing antibody binding based only on absorbance values. In summary, these data have confirmed that a cell-based ELISA with an antigen-decorated cell surface is an alternative, fast, and easy adaptive approach to detecting IgG. The advantage is that the antigen is expressed by the cells themselves, which makes it easy to test several antigens on one plate without the need for protein isolation and purification steps. Especially, when any antigen design must be optimized, a cell-based approach is very useful for the initial screening of different antigen designs. We are convinced that our developed tANCHOR cell-based ELISA approach is applicable to other antigens of different pathogens for use in serological analysis or to determine the immunization status of patients.

## Limitations and prerequisites

A critical factor that may influence the outcome of the assay is the purity and amount of plasmid DNA used when comparing variants. Plasmid DNA has exceptional stability compared with other biological materials, such as proteins [57]. The use of the same bacterial strain and plasmid isolation kits will increase the reproducibility of results obtained from cell-based ELISA. Notably, we did not observe any mislocalization or drastic differences in protein expression levels between the Wuhan, Delta, and Omicron RBD variants (Figs. 2B–D). Prior to serum testing, the protein localization and expression of each emerging SARS-CoV-2 RBD variant need to be verified. This consideration is relevant because an emerging variant could possibly cause an altered expression when fused to the tANCHOR system. Since similar effects have not yet been observed for other proteins displayed using the tANCHOR display technology, we can only presume that further SARS-CoV-2 RBD variants will likewise be displayed on the cell surface, as this study has shown [24, 27, 48, 58]. Antibody-binding capability is another significant drawback of this kind of developed assay. Certain antibodies that can bind to the RBD may be lost as a result of inadequate cell transfection, which will reduce the binding values. Thus, achieving high transfection efficiency is crucial for reliable results. In addition to Metafectene, other transfection reagents such as Jetprime (PolyPlus, Illkirch, France) or HeLaFect (OZ Biosciences SAS, Marseille, France) may be employed. The C-terminally fused reporter protein mCherry makes it simple to check the transfection efficiency when optimizing the transfection parameters with other reagents. Another crucial factor to consider is the prevalence of mycoplasma contamination in laboratory cell cultures [59]. Between 5% and 35% of cell cultures are contaminated with mycoplasma species like *Acholeplasma laidlawii*, *Mycoplasma arginini*, *M. fermentans*, *M. hyorhinis*, and *M. orale* [60, 61]. The fact that human serum contains anti-Mycoplasma spp. IgG demands that cultivated HeLa cells are mycoplasma-free [62]. Otherwise, IgG from humans could detect such antigens, resulting in false-positive antigen-specific binding to the tANCHORed protein.

## Ethical statement

This study was conducted in accordance with the ethical guidelines of the 1975 Declaration of Helsinki. The study was conducted according to federal guidelines and local ethics committee regulations (Albert-Ludwigs-Universität, Freiburg, Germany: Nos. F-2020-09-03-160428, 322/20, 20-1271_1), and it received approval from the institutional ethical committee of the University of Freiburg (EK 153/20). Written informed consent was obtained from the study participants, and these participants did not receive any compensation.

## Supplementary Material

bpae001_Supplementary_Data

## Data Availability

The data that support the findings of this study are available from the corresponding author upon reasonable request.

## References

[1] Wang C , Horby PW, Hayden FG et al A novel coronavirus outbreak of global health concern. Lancet 2020;395:470–3. 31986257 10.1016/S0140-6736(20)30185-9 PMC7135038

[2] Phelan AL , Katz R, Gostin LO. The novel coronavirus originating in Wuhan, China: challenges for global health governance. Jama 2020;323:709–10. 31999307 10.1001/jama.2020.1097

[3] Li X , Zai J, Wang X et al Potential of large “first generation” human-to-human transmission of 2019-nCoV. J Med Virol 2020;92:448–54. 31997390 10.1002/jmv.25693 PMC7166825

[4] Ji W , Wang W, Zhao X et al Cross-species transmission of the newly identified coronavirus 2019-nCoV. J Med Virol 2020;92:433–40. 31967321 10.1002/jmv.25682 PMC7138088

[5] Huang C , Wang Y, Li X et al Clinical features of patients infected with 2019 novel coronavirus in Wuhan, China. Lancet 2020;395:497–506. 31986264 10.1016/S0140-6736(20)30183-5 PMC7159299

[6] Ke Z , Oton J, Qu K et al Structures and distributions of SARS-CoV-2 spike proteins on intact virions. Nature 2020;588:498–502. 32805734 10.1038/s41586-020-2665-2 PMC7116492

[7] Kim D , Lee JY, Yang JS et al The architecture of SARS-CoV-2 transcriptome. Cell 2020;181:914–21.e10. 32330414 10.1016/j.cell.2020.04.011 PMC7179501

[8] Xiaojie S , Yu L, Lei Y et al Neutralizing antibodies targeting SARS-CoV-2 spike protein. Stem Cell Res 2020;50:102125. 33341604 10.1016/j.scr.2020.102125 PMC7737530

[9] Yadav T , Srivastava N, Mishra G et al Recombinant vaccines for COVID-19. Hum Vaccin Immunother 2020;16:2905–12. 33232211 10.1080/21645515.2020.1820808 PMC7711739

[10] Yan R , Zhang Y, Li Y et al Structural basis for the recognition of SARS-CoV-2 by full-length human ACE2. Science 2020;367:1444–8. 32132184 10.1126/science.abb2762 PMC7164635

[11] Yang J , Wang W, Chen Z et al A vaccine targeting the RBD of the S protein of SARS-CoV-2 induces protective immunity. Nature 2020;586:572–7. 32726802 10.1038/s41586-020-2599-8

[12] Niu L , Wittrock KN, Clabaugh GC et al A structural landscape of neutralizing antibodies against SARS-CoV-2 receptor binding domain. Front Immunol 2021;12:647934. 33995366 10.3389/fimmu.2021.647934 PMC8113771

[13] Bloom JD , Beichman AC, Neher RA et al Evolution of the SARS-CoV-2 mutational spectrum. Mol Biol Evol 2023;40.Apr 4;40(4):msad085. 10.1093/molbev/msad085 PMC10124870 37039557

[14] Peck KM , Lauring AS. Complexities of viral mutation rates. J Virol 2018;92.Jun 29;92(14):e01031–17. 29720522 10.1128/JVI.01031-17 PMC6026756

[15] Kudriavtsev AV , Vakhrusheva AV, Novosеletsky VN et al Immune escape associated with RBD Omicron mutations and SARS-CoV-2 evolution dynamics. Viruses 2022;14.Jul 22;14(8):1603. 10.3390/v14081603 PMC9394476 35893668

[16] Chakraborty C , Bhattacharya M, Sharma AR et al Omicron (B.1.1.529) - a new heavily mutated variant: mapped location and probable properties of its mutations with an emphasis on S-glycoprotein. Int J Biol Macromol 2022;219:980–97. 35952818 10.1016/j.ijbiomac.2022.07.254 PMC9359758

[17] Thakur V , Ratho RK. OMICRON (B.1.1.529): a new SARS-CoV-2 variant of concern mounting worldwide fear. J Med Virol 2022;94:1821–4. 34936120 10.1002/jmv.27541

[18] Ladner JT , Henson SN, Boyle AS et al Epitope-resolved profiling of the SARS-CoV-2 antibody response identifies cross-reactivity with endemic human coronaviruses. Cell Rep Med 2021;2:100189. 33495758 10.1016/j.xcrm.2020.100189 PMC7816965

[19] Premkumar L , Segovia-Chumbez B, Jadi R et al The receptor binding domain of the viral spike protein is an immunodominant and highly specific target of antibodies in SARS-CoV-2 patients. Sci Immunol 2020;5:10.1126/sciimmunol.abc8413 PMC7292505 32527802

[20] Padoan A , Cosma C, Della Rocca F et al A cohort analysis of SARS-CoV-2 anti-spike protein receptor binding domain (RBD) IgG levels and neutralizing antibodies in fully vaccinated healthcare workers. Clin Chem Lab Med 2022;60:1110–5. 35473824 10.1515/cclm-2022-0322

[21] Swadźba J , Anyszek T, Panek A et al Head-to-head comparison of 5 anti-SARS-CoV-2 assays performance in one hundred COVID-19 vaccinees, over an 8-month course. Diagnostics (Basel) 2022;12.Jun 9;12(6):1426. 10.3390/diagnostics12061426 PMC9221713 35741236

[22] Müller L , Kannenberg J, Biemann R et al Comparison of the measured values of quantitative SARS-CoV-2 spike antibody assays. J Clin Virol 2022;155:105269. 36029637 10.1016/j.jcv.2022.105269 PMC9388276

[23] Ivanusic D , Madela K, Burghard H et al tANCHOR: a novel mammalian cell surface peptide display system. Biotechniques 2021;70:21–28. 33307791 10.2144/btn-2020-0073

[24] Ivanusic D , Pietsch H, Konig J et al Absence of IL-10 production by human PBMCs co-cultivated with human cells expressing or secreting retroviral immunosuppressive domains. PLoS One 2018;13:e0200570. 30001404 10.1371/journal.pone.0200570 PMC6042780

[25] Khare S , Gurry C, Freitas L et al GISAID‘s role in pandemic response. China CDC Wkly 2021;3:1049–51. 34934514 10.46234/ccdcw2021.255 PMC8668406

[26] Ivanusic D , Madela K, Bannert N et al The large extracellular loop of CD63 interacts with gp41 of HIV-1 and is essential for establishing the virological synapse. Sci Rep 2021;11:10011. 33976357 10.1038/s41598-021-89523-7 PMC8113602

[27] Ivanusic D , Denner J. The large extracellular loop is important for recruiting CD63 to exosomes. MicroPubl Biol 2023;2023:10.17912/micropub.biology.000842 PMC10432940 37602284

[28] Piña R , Santos-Díaz AI, Orta-Salazar E et al Ten approaches that improve immunostaining: a review of the latest advances for the optimization of immunofluorescence. Int J Mol Sci 2022;23.Jan 26;23(3):1426. 10.3390/ijms23031426 PMC8836139 35163349

[29] Boassa D. Correlative microscopy for localization of proteins in situ: pre-embedding immuno-electron microscopy using fluoronanogold, gold enhancement, and low-temperature resin. Methods Mol Biol 2015;1318:173–80. 26160575 10.1007/978-1-4939-2742-5_17 PMC5716471

[30] Hoffman EA , Frey BL, Smith LM et al Formaldehyde crosslinking: a tool for the study of chromatin complexes. J Biol Chem 2015;290:26404–11. 26354429 10.1074/jbc.R115.651679 PMC4646298

[31] Demmer RT , Baumgartner B, Wiggen TD et al Identification of natural SARS-CoV-2 infection in seroprevalence studies among vaccinated populations. Mayo Clin Proc 2022;97:754–60. 35379422 10.1016/j.mayocp.2022.02.002 PMC8841164

[32] Raoufi E , Hosseini F, Onagh B et al Designing and developing a sensitive and specific SARS-CoV-2 RBD IgG detection kit for identifying positive human samples. Clin Chim Acta 2023;542:117279. 36871661 10.1016/j.cca.2023.117279 PMC9985519

[33] Hayrapetyan H , Tran T, Tellez-Corrales E et al Enzyme-linked immunosorbent assay: types and applications. Methods Mol Biol 2023;2612:1–17. 36795355 10.1007/978-1-0716-2903-1_1

[34] Sarngadharan MG , DeVico AL, Bruch L et al HTLV-III: the etiologic agent of AIDS. Princess Takamatsu Symp 1984;15:301–8. 6100648

[35] Guo L , Ren L, Yang S et al Profiling early humoral response to diagnose novel coronavirus disease (COVID-19). Clin Infect Dis 2020;71:778–85. 32198501 10.1093/cid/ciaa310 PMC7184472

[36] Schramm W , Roggendorf M, Rommel F et al Prevalence of antibodies to hepatitis C virus (HCV) in haemophiliacs. Blut 1989;59:390–2. 2506955 10.1007/BF00321210

[37] Sansonno D , Vacca A, Gernone A et al HBeAg/anti-HBe circulating immune complexes in patients chronically infected with hepatitis B virus. Ric Clin Lab 1989;19:81–91. 2762731 10.1007/BF02871796

[38] Veldkamp J , Visser AM. Application of the enzyme-linked immunosorbent assay (ELISA) in the serodiagnosis of syphilis. Br J Vener Dis 1975;51:227–31. 1098730 10.1136/sti.51.4.227 PMC1046555

[39] Nassau E , Parsons ER, Johnson GD. The detection of antibodies to Mycobacterium tuberculosis by microplate enzyme-linked immunosorbent assay (ELISA). Tubercle 1976;57:67–70. 821196 10.1016/0041-3879(76)90019-2

[40] Tcherniaeva I , den Hartog G, Berbers G et al The development of a bead-based multiplex immunoassay for the detection of IgG antibodies to CMV and EBV. J Immunol Methods 2018;462:1–8. 30056034 10.1016/j.jim.2018.07.003

[41] Richalet-Sécordel PM , Van Regenmortel MH. A new capture test using conjugated peptides for the detection of HIV antibodies. FEMS Microbiol Immunol 1991;4:57–64. 1815712 10.1111/j.1574-6968.1991.tb04971.x

[42] Tai W , He L, Zhang X et al Characterization of the receptor-binding domain (RBD) of 2019 novel coronavirus: implication for development of RBD protein as a viral attachment inhibitor and vaccine. Cell Mol Immunol 2020;17:613–20. 32203189 10.1038/s41423-020-0400-4 PMC7091888

[43] Rahbar Z , Nazarian S, Dorostkar R et al Recombinant expression of SARS-CoV-2 receptor binding domain (RBD) in Escherichia coli and its immunogenicity in mice. Iran J Basic Med Sci 2022;25:1110–6. 36246069 10.22038/IJBMS.2022.65045.14333 PMC9526882

[44] Wagner A , Guzek A, Ruff J et al Neutralising SARS-CoV-2 RBD-specific antibodies persist for at least six months independently of symptoms in adults. Commun Med (Lond) 2021;1:13. 35602189 10.1038/s43856-021-00012-4 PMC9037317

[45] Elena SF , Sanjuán R. Adaptive value of high mutation rates of RNA viruses: separating causes from consequences. J Virol 2005;79:11555–8. 16140732 10.1128/JVI.79.18.11555-11558.2005 PMC1212614

[46] Cao Y , Wang J, Jian F et al Omicron escapes the majority of existing SARS-CoV-2 neutralizing antibodies. Nature 2022;602:657–63. 35016194 10.1038/s41586-021-04385-3 PMC8866119

[47] Kaku CI , Starr TN, Zhou P et al Evolution of antibody immunity following Omicron BA.1 breakthrough infection. Nat Commun 2023;14:2751. 37173311 10.1038/s41467-023-38345-4 PMC10180619

[48] Bernauer H , Schlor A, Maier J et al tANCHOR fast and cost-effective cell-based immunization approach with focus on the receptor-binding domain of SARS-CoV-2. Biol Methods Protoc 2023;8:bpad030. 38090673 10.1093/biomethods/bpad030 PMC10713279

[49] Barlow DJ , Edwards MS, Thornton JM. Continuous and discontinuous protein antigenic determinants. Nature 1986;322:747–8. 2427953 10.1038/322747a0

[50] Benjamin DC , Berzofsky JA, East IJ et al The antigenic structure of proteins: a reappraisal. Annu Rev Immunol 1984;2:67–101. 6085753 10.1146/annurev.iy.02.040184.000435

[51] Ivanusic D , Eschricht M, Denner J. Investigation of membrane protein-protein interactions using correlative FRET-PLA. BioTechniques 2014;57:188–98. 25312088 10.2144/000114215

[52] Ivanusic D. HIV-1 cell-to-cell spread: CD63-gp41 interaction at the virological synapse. AIDS Res Hum Retroviruses 2014;30:844–5. 25184547 10.1089/aid.2014.0116

[53] Ivanusic D , Heinisch JJ, Eschricht M et al Improved split-ubiquitin screening technique to identify surface membrane protein-protein interactions. BioTechniques 2015;59:63–73. 26260084 10.2144/000114315

[54] Pi-Estopinan F , Perez MT, Fraga A et al A cell-based ELISA as surrogate of virus neutralization assay for RBD SARS-CoV-2 specific antibodies. Vaccine 2022;40:1958–67. 35193792 10.1016/j.vaccine.2022.02.044 PMC8856731

[55] Ruetalo N , Flehmig B, Schindler M et al Long-term humoral immune response against SARS-CoV-2 after natural infection and subsequent vaccination according to WHO International binding antibody units (BAU/mL). Viruses 2021;13. Nov 23;13(12):2336. 10.3390/v13122336 PMC8708153 34960605

[56] Knezevic I , Mattiuzzo G, Page M et al WHO International Standard for evaluation of the antibody response to COVID-19 vaccines: call for urgent action by the scientific community. Lancet Microbe 2022;3:e235–e240. 34723229 10.1016/S2666-5247(21)00266-4 PMC8547804

[57] Nguyen HH , Park J, Park SJ et al Long-term stability and integrity of plasmid-based DNA data storage. Polymers (Basel) 2018;10.Jan 1;10(1):28. 10.3390/polym10010028 PMC6415062 30966073

[58] Ivanusic D , Madela K, Bannert N et al Time-lapse imaging of CD63 dynamics at the HIV-1 virological synapse by using agar pads. MicroPubl Biol 2022;2022.Oct 7:2022. 10.17912/micropub.biology.000648. PMC9587459 36281316

[59] Drexler HG , Uphoff CC. Mycoplasma contamination of cell cultures: incidence, sources, effects, detection, elimination, prevention. Cytotechnology 2002;39:75–90. 19003295 10.1023/A:1022913015916 PMC3463982

[60] Hay RJ , Macy ML, Chen TR. Mycoplasma infection of cultured cells. Nature 1989;339:487–8. 2725683 10.1038/339487a0

[61] Nikfarjam L , Farzaneh P. Prevention and detection of Mycoplasma contamination in cell culture. Cell J 2012;13:203–12. 23508237 PMC3584481

[62] Arfi Y , Lartigue C, Sirand-Pugnet P et al Beware of mycoplasma anti-immunoglobulin strategies. mBio 2021;12:e0197421. 34781733 10.1128/mBio.01974-21 PMC8593674

