# Supplementary material for: tANCHOR cell-based ELISA approach as a surrogate for antigen-coated plates to monitor specific IgG directed to the SARS-CoV-2 receptor-binding domain
Source: Biol Methods Protoc. 2024 Jan 19;9(1):bpae001. doi: 10.1093/biomethods/bpae001 (PMC10850845; doi:10.1093/biomethods/bpae001)
Supplement: bpae001_Supplementary_Data [file bpae001_supplementary_data.docx]

**Supplementary Information**

**tANCHOR cell-based ELISA approach as a surrogate for antigen-coated plates to monitor specific IgG directed to the SARS-CoV-2 receptor binding domain**

Bernauer, Hubert; Maier, Josef; Bannert, Norbert and Ivanusic, Daniel

**Supplementary Table 1**

| SARS-CoV-2 Wuhan-Hu-1 RBD gene synthesis fragment  GenBank accession: NC_045512.2:22517-23191 |
| --- |
| GAATTCAGAGTCCAACCAACAGAATCTATTGTTAGATTTCCTAATATTACAAACTTGTGCCCTTTTGGTGAAGTTTTTAACGCCACCAGATTTGCATCTGTTTATGCTTGGAACAGGAAGAGAATCAGCAACTGTGTTGCTGATTATTCTGTCCTATATAATTCCGCATCATTTTCCACTTTTAAGTGTTATGGAGTGTCTCCTACTAAATTAAATGATCTCTGCTTTACTAATGTCTATGCAGATTCATTTGTAATTAGAGGTGATGAAGTCAGACAAATCGCTCCAGGGCAAACTGGAAAGATTGCTGATTATAATTATAAATTACCAGATGATTTTACAGGCTGCGTTATAGCTTGGAACTCTAACAATCTTGATTCTAAGGTTGGTGGTAATTATAATTACCTGTATAGATTGTTTAGGAAGTCTAATCTCAAACCTTTTGAGAGAGATATTTCAACTGAAATCTATCAGGCCGGTAGCACACCTTGTAATGGTGTTGAAGGTTTTAATTGTTACTTTCCTTTACAATCATATGGTTTCCAACCCACTAATGGTGTTGGTTACCAACCATACAGAGTAGTAGTACTTTCTTTTGAACTTCTACATGCACCAGCAACTGTTTGTGGACCTAAAAAGTCTACTAATTTGGTTAAAAACAAATGTGTCAATTTCAACTTCGATATC |
| SARS-CoV-2 Wuhan-Hu-1 RBD protein sequence GenBank accession: YP_009724390.1:318-543 |
| EFRVQPTESIVRFPNITNLCPFGEVFNATRFASVYAWNRKRISNCVADYSVLYNSASFSTFKCYGVSPTKLNDLCFTNVYADSFVIRGDEVRQIAPGQTGKIADYNYKLPDDFTGCVIAWNSNNLDSKVGGNYNYLYRLFRKSNLKPFERDISTEIYQAGSTPCNGVEGFNCYFPLQSYGFQPTNGVGYQPYRVVVLSFELLHAPATVCGPKKSTNLVKNKCVNFNFDI |
| Note: Underlined sequence contains the flanked restriction sites *Eco*RI and *Eco*RV. *Eco*RI is coding for EF and therefore F is part of the RBD that is corresponding to amino acid position 318 GenBank: YP_009724390.1. |
| \| **Obtained sequence for construction of Delta and Omicron RBD variants**  **Data Availability at**  GISAID Identifier: EPI_SET_230215my  doi: 10.55876/gis8.230215my  All genome sequences and associated metadata in this dataset are published in GISAID’s EpiCoV database. To view the contributors of each individual sequence with details such as accession number, Virus name, Collection date, Originating Lab and Submitting Lab and the list of Authors, visit [10.55876/gis8.230215my](https://epicov.org/epi3/epi_set/230215my)  **Data Snapshot** EPI_SET_230215my is composed of 9 individual genome sequences. The collection dates range from 2019-12-30 to 2021-11-20; Data were collected in 7 countries and territories; All sequences in this dataset are compared relative to hCoV-19/Wuhan/WIV04/2019 (WIV04), the official reference sequence employed by GISAID (EPI_ISL_402124). Learn more at <https://gisaid.org/WIV04>**.** \| \| \| \| \| \| --- \| --- \| --- \| --- \| --- \| \|  \|  \|  \| **Position in genome** \| \| \| **SARS-CoV-2 variant** \| **GISAID EPI_ISL** \| **Strain** \| **Start** \| **End** \| \| Wuhan-Hu-1 \| EPI_ISL_402124.1 \| hCoV-19/Wuhan/WIV04/2019 \| 22517 \| 23191 \| \| Delta B.1.617.2 \| EPI_ISL_2378732.1 \| hCoV-19/Japan/TKYTK1734/2021 \| 22507 \| 23181 \| \| Omicron BA.1 \| EPI_ISL_6640916.1 \| hCoV-19/Botswana/R40B59_BHP_3321001248/2021 \| 22442 \| 23116 \| \| Omicron BA.1 \| EPI_ISL_6704867.1 \| hCoV-19/South_Africa/NICD-N21668/2021 \| 22493 \| 23167 \| \|  \|  \|  \|  \|  \| \| Note: The RBD coding sequence is 675 base pairs long and encodes 225 amino acids. It represents the amino acids 319-543 of the spike protein (according to Wuhan-1 numbering). For Omicron BA-1, two type strains were respectively considered with the same outcome. The RBD coding sequences of Omicron BA.1 strains are identical to each other, respectively. The *Eco*RI restriction recognition sites at position 354-359 GAATTC, with positions counted from the start of codon F318, present within the RBD-coding sequences in all strains, were removed by silent base changes 357TC. N-terminal *Eco*RI is coding for EF and therefore F is part of the RBD that is corresponding to aa position 318.   \| **Variant** \| **Mutations within the RBD** \| \| --- \| --- \| \| Delta B.1.617.2 \| L452R, T478K \| \| Omicron BA.1 \| G339D, S371L, S373P, S375F, K417N, N440K, G446S, S477N, T478K, E484A, Q493R, G496S, Q498R, N501Y, Y505H \| \| \| \| \| \| |

**Supplementary Table 2**

| **Primer** | **DNA Sequence 5´🡪3´** |
| --- | --- |
| YFP-*Cla*I for | TTTTTAAGCTTATCGATGTGAGCAAGGGCGAGGAGCTG |
| YFP-*Pme*I | TTTTTGTTTAAACTCATGATCACTTGTACAGCTCGTCCATG |

**Supplementary Protocols**

**Protocol for Sanger sequencing**

Plasmid identity was checked by Sanger sequencing using the BigDye Terminator v3.1 cycle Sequencing Kit (PE Applied Biosystems, Darmstadt, Germany). To set up PCR reaction a mix of 250 ng of plasmid DNA, 0.5 μL of 10 μM diluted forward primer AACGAGGTCCGCTGCCTG (Integrated DNA Technologies, Leuven, Belgium), 2 μL of 5X sequencing buffer, and 0.5 μL of BigDye Terminator 3.1, filled with sterile, deionized water to a total volume of 10 μL was prepared. PCR mixture was initially denatured for 1 min at 96°C, followed by 25 cycles for amplification at 96°C for 10 sec, 54°C for 15 sec, and 60°C for 4 min. The readout of DNA sequence was performed by separation of PCR products via capillary electrophoresis using an automated DNA sequencer (ABI-Prism 3100 Genetic Analyzer, Applied Biosystems).

**Protocol for isolation of plasmid DNA using QIAGEN Plasmid Maxi Kit**

Plasmid DNA form the *E. coli* strain DH5Alpha was isolated by following protocol.

1. Harvest overnight bacterial culture by centrifuging at 6000 x g for 15 min at 4°C.

2. Resuspend the bacterial pellet in 10 mL Buffer P1.

3. Add 10 mL Buffer P2, mix thoroughly by vigorously inverting 4–6 times and incubate at room temperature (15–25°C) for 5 min. If using LyseBlue reagent, the solution will turn blue.

4. Add 10 mL prechilled Buffer P3, mix thoroughly by vigorously inverting 4–6 times. Incubate on ice for 20 min. If using LyseBlue reagent, mix the solution until it is colorless.

5. Centrifuge at ≥20,000 x g for 30 min at 4°C. Re-centrifuge the supernatant at ≥20,000 x g for 15 min at 4°C.

6. Equilibrate a QIAGEN-tip 500 by applying 10 mL Buffer QBT on the column.

7. Apply the supernatant from step 5 to the QIAGEN-tip.

8. Wash the QIAGEN-tip with 2 x 30 mL Buffer QC.

9. Elute DNA with 15 mL Buffer QF into a clean 50 mL conical centrifuge tube (TPP, Trasadingen, Switzerland).

10. Precipitate DNA by adding 10.5 mL of isopropanol to the eluted DNA and mix. Centrifuge at ≥15,000 x g for 30 min at 4°C. Carefully decant the supernatant.

11. Wash the DNA pellet with 5 mL 70% ethanol and centrifuge at ≥15,000 x g for 10 min. Carefully decant supernatant.

12. Air-dry pellet for 5–10 min and dissolve DNA in 200 µL sterile, deionized water.

**Protocol for cell transfection mix**

For one 96 well or one Ibidi μ-slide well the transfection mix was prepared in the following way:

**Solution 1:** 0.3 μg of plasmid DNA was added to 50 μL serum-free DMEM.

**Solution 2:** 1 μL of Metafectene transfection reagent (Biontex, Munich, Germany) was added to 50 μL serum-free DMEM.

Medium was pipetted first before plasmid DNA or transfection reagent was added to the tube.

Dulbecco's Modified Eagle Medium (DMEM) was supplemented with 2 mM L-glutamine, 100 U mL^-1^ penicillin, and 100 µg mL^-1^ streptomycin.

Solution **1** and **2** were mixed carefully by pipetting one time and incubated for 30 min at room temperature before being added to the cells.
